# Signs of Neutralization in a Redundant Gene Involved in Homologous Recombination in *Wolbachia* Endosymbionts

**DOI:** 10.1093/gbe/evu207

**Published:** 2014-09-17

**Authors:** Myriam Badawi, Isabelle Giraud, Fabrice Vavre, Pierre Grève, Richard Cordaux

**Affiliations:** ^1^Université de Poitiers, UMR CNRS 7267 Ecologie et Biologie des Interactions, Equipe Ecologie Evolution Symbiose, Poitiers, France; ^2^Université de Lyon, UMR CNRS 5558 Biométrie et Biologie Evolutive, Villeurbanne, France

**Keywords:** endosymbiosis, gene loss, molecular evolution, selection relaxation, genomic reduction, nonorthologous gene displacement

## Abstract

Genomic reduction in bacterial endosymbionts occurs through large genomic deletions and long-term accumulation of mutations. The latter process involves successive steps including gene neutralization, pseudogenization, and gradual erosion until complete loss. Although many examples of pseudogenes at various levels of degradation have been reported, neutralization cases are scarce because of the transient nature of the process. Gene neutralization may occur due to relaxation of selection in nonessential genes, for example, those involved in redundant functions. Here, we report an example of gene neutralization in the homologous recombination (HR) pathway of *Wolbachia,* a bacterial endosymbiont of arthropods and nematodes. The HR pathway is often depleted in endosymbiont genomes, but it is apparently intact in some *Wolbachia* strains. Analysis of 12 major HR genes showed that they have been globally under strong purifying selection during the evolution of *Wolbachia* strains hosted by arthropods, supporting the evolutionary importance of the HR pathway for these *Wolbachia* genomes. However, we detected signs of recent neutralization of the *ruvA* gene in a subset of *Wolbachia* strains, which might be related to an ancestral, clade-specific amino acid change that impaired DNA-binding activity. Strikingly, RuvA is part of the RuvAB complex involved in branch migration, whose function overlaps with the RecG helicase. Although *ruvA* is experiencing neutralization, *recG* is under strong purifying selection. Thus, our high phylogenetic resolution suggests that we identified a rare example of targeted neutralization of a gene involved in a redundant function in an endosymbiont genome.

## Introduction

**Fig. 1.—:**
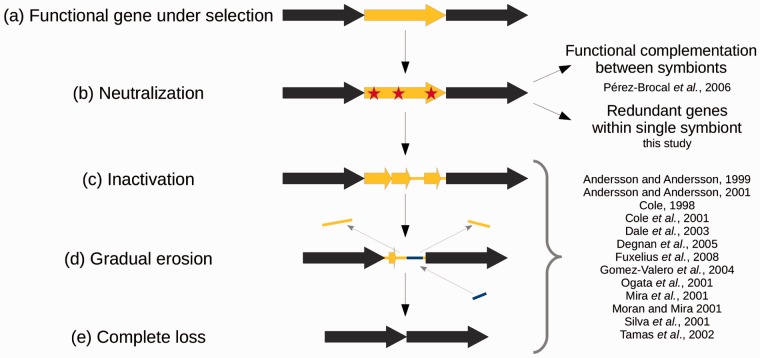
Gene loss through pseudogenization. Starting from a functional gene under selection (*a*) relaxation of selective pressures leads to accumulation of slightly deleterious mutations and neutralization of the gene (*b*) until the gene is effectively inactivated and pseudogenized (*c*). A bias in favor of deletions (yellow bars) relative to insertions (blue bars) leads to gradual erosion of the pseudogene (*d*) until its complete loss (*e*).

Host-restricted intracellular bacteria, either as parasites, commensals or mutualistic symbionts, exhibit multiple distinguishing genomic features in comparison with their free-living relatives. Hence, obligate bacterial endosymbionts (that live and replicate exclusively in the cytoplasm of the host cells) are characterized by reduced genomes, accelerated DNA sequence evolution, and strong A+T nucleotide compositional bias (Wernegreen 2005; Moran et al. 2008; Moya et al. 2008; McCutcheon and Moran 2012; Van Leuven and McCutcheon 2012). These features are the consequences of the process of genomic reduction, which is triggered by enhanced genetic drift and relaxation of selection because of effective population size reduction and stable environmental conditions (Moran 1996; Nilsson et al. 2005; Toft and Andersson 2010). The initial stages are characterized by large genomic deletions mediated by recombination between proliferating mobile genetic elements (Moran and Plague 2004; Plague et al. 2008; Walker and Langridge 2008; Cerveau, Leclercq, Bouchon, et al. 2011). On the long term, relaxed purifying selection leads to the accumulation of slightly deleterious mutations and the inactivation of nonessential genes or genes with redundant functions (Ohta and Gillespie 1996; Silva et al. 2001; Tamas et al. 2002; Dagan et al. 2006; Moran et al. 2009) (fig. 1). Coupled with a deletion bias, newly formed pseudogenes are ultimately lost along with mobile genetic elements (Mira et al. 2001; Moran and Mira 2001; Ogata et al. 2001; Gómez-Valero et al. 2004; Fuxelius et al. 2008) (fig. 1). In addition, gene loss can involve genes carrying essential functions such as DNA repair, which further increases the rate of gene loss (Dale et al. 2003; Rocha et al. 2005). In particular, genes involved in the homologous recombination (HR) pathway are often depleted in these genomes, implicating fewer recombination events (Dale et al. 2003; Rocha et al. 2005; Moran et al. 2008). This is consistent with the long-term genomic stability observed in various ancient endosymbiont genomes (Tamas et al. 2002; Silva et al. 2003). Although the later stages of gene loss in endosymbionts (from gene inactivation to complete loss) have been well-documented (fig. 1 and references therein), few studies have described examples of the initial step of gene loss, that is, neutralization preceding inactivation. This is because most analyzed genomes are from ancient endosymbionts with highly reduced genomes, in which the process of gene loss is already at an advanced stage.

Various mechanisms can lead to gene neutralization in endosymbiont genomes, either by altering all genes of the genome in the same manner or by targeting specific genes. In the first case, enhanced genetic drift and less efficient purifying selection, coupled with a Muller’s ratchet effect due to reduced opportunities for recombination, lead to the global accumulation of slightly deleterious mutations in many genes. This scenario explains the general acceleration in DNA sequence evolution observed in endosymbiont genomes (Moran 1996; Ohta and Gillespie 1996; Wernegreen and Moran 1999; Itoh et al. 2002; Woolfit and Bromham 2003; Wernegreen and Funk 2004; Fry and Wernegreen 2005; Blanc et al. 2007; McCutcheon and Moran 2012). Gene neutralization can also occur due to targeted relaxation of selection on a gene that became superfluous for the endosymbiont, as in the case in which a gene is involved in a redundant function with another gene (Moran et al. 2008; Moya et al. 2008). This is well illustrated by functional complementation, which may happen when multiple endosymbionts co-occurring within a single host can fulfill the same function. This has been demonstrated for the endosymbionts *Buchnera aphidicola* BCc and *Serratia symbiotica* in the insect *Cinara cedri*, in which *B. aphidicola* is undergoing genome degradation and functional replacement by the coexisting *S. symbiotica* (Pérez-Brocal et al. 2006). Alternatively, functional redundancy may occur within a single endosymbiont genome. However, to our knowledge, there has been no report clearly demonstrating targeted relaxation of selective pressures in such functionally redundant genes leading to neutralization. This is because gene neutralization is a transient stage preceding actual pseudogenization, whose detection requires a high phylogenetic resolution and, thus, investigation of a large set of closely related endosymbiont strains.

In this study, we report an example of likely gene neutralization in a redundant portion of the HR pathway within the bacterial endosymbiont *Wolbachia*. *Wolbachia* are maternally inherited microorganisms that have been associated with arthropod and nematode hosts for greater than 100 Myr and are able to manipulate arthropod host reproduction to increase their own transmission (Bandi et al. 1998; Werren et al. 2008; Cordaux et al. 2011). *Wolbachia* endosymbionts present a large genetic diversity, with multiple phylogenetic supergroups defined with capital letters (Lo et al. 2007). In particular, *Wolbachia* strains from supergroups A and B are found in arthropods and *Wolbachia* strains from supergroups C and D are found in nematodes. Multiple *Wolbachia* genomes have been sequenced; they show typical features of long-term obligate endosymbionts, such as reduced genome size, accelerated DNA sequence evolution, and A+T nucleotide bias (Wu et al. 2004; Foster et al. 2005; Klasson et al. 2008; Klasson et al. 2009; Darby et al. 2012). Yet, despite their ancient association with invertebrates, many *Wolbachia* genomes contain recently active mobile genetic elements (Cordaux et al. 2008; Kent and Bordenstein 2010; Cerveau, Leclercq, Leroy, et al. 2011; Leclercq et al. 2011). In addition, several *Wolbachia* strains experience recombination (Werren and Bartos 2001; Ellegaard et al. 2013) and gene conversion (Cordaux 2009). Strikingly, the HR pathway, which is commonly depleted in long-term endosymbionts (Akman et al. 2002; Tamas et al. 2002; Dale et al. 2003; Gil et al. 2003; Rocha et al. 2005), is apparently intact in some (Wu et al. 2004; Foster et al. 2005), but not all (Darby et al. 2012), *Wolbachia* genomes.

**Fig. 2.—:**
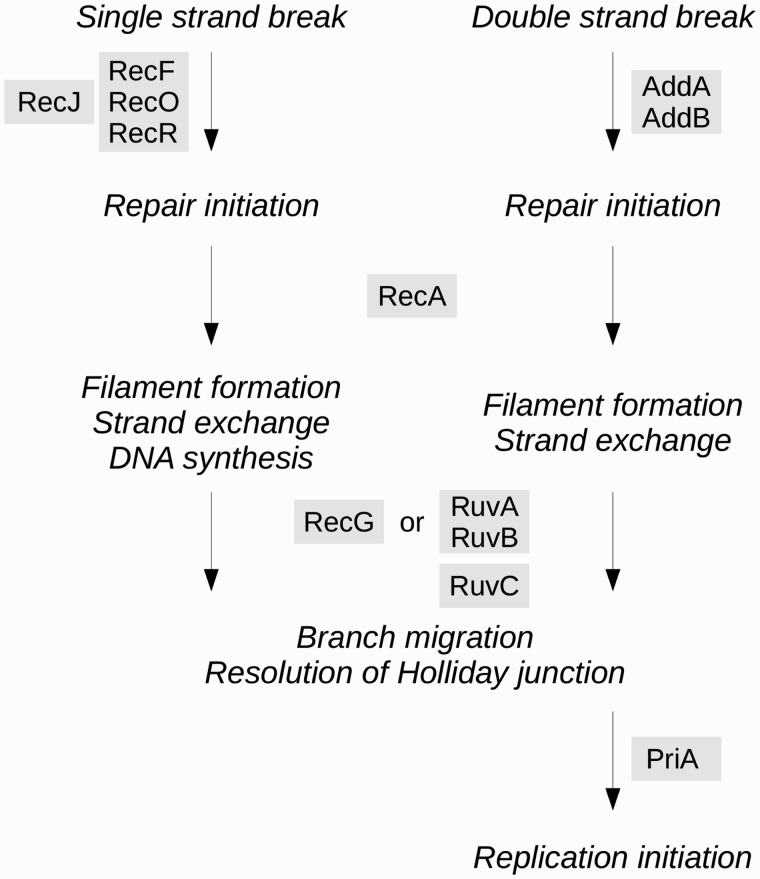
Schematic representation of the HR pathway. Repair of DNA single- and double-strand breaks is initiated by RecJ/RecFOR and AddAB, respectively. The initiation step promotes the recruitment of RecA filament that catalyzes strand exchange. Then, branch migration and resolution of Holliday junction is completed by the RuvABC complex or RecG helicase. Repair of DNA double-strand breaks is finally completed with PriA which mediates primosome assembly for replication.

The HR pathway is involved in DNA repair of single- and double-strand breaks and is responsible for large-scale genomic rearrangements and incorporation of homologous foreign DNA (Aravind et al. 2000; Kowalczykowski 2000; Zuñiga-Castillo et al. 2004) (fig. 2). Interestingly, a crucial step of the HR pathway (branch migration) may be fulfilled by genes with overlapping functions, that is, the RuvAB complex or the RecG helicase, although efficiency is reduced when only one of the two possibilities is active (Meddows et al. 2004). In the context of genomic reduction undergone by *Wolbachia*, such functional redundancy is predicted to be dispensable (Mendonça et al. 2011). These observations raise the question whether the HR pathway in general and functional redundancy in this pathway in particular are maintained by purifying selection in *Wolbachia* because they are essential, or HR happens to take place merely because the pathway has not been pseudogenized yet. To address this question, we assessed the distribution of 12 major genes of the HR pathway in 20 sequenced genomes from the four major *Wolbachia* supergroups A–D (Lo et al. 2007; Comandatore et al. 2013). This prompted us to analyze selection patterns of HR genes in *Wolbachia* supergroups A and B, using an extended set of *Wolbachia* strains comprising both sequenced genomes and strains for which we performed targeted resequencing. This high phylogenetic resolution enabled us to uncover signs of recent neutralization of the redundant *ruvA* gene in a subset of *Wolbachia* strains from supergroup B hosted by isopods.

## Materials and Methods

### Identification of HR genes

We selected 12 genes encoding the major proteins predicted to be involved in the HR pathway in *Wolbachia* (Aravind et al. 2000; Kowalczykowski 2000; Zuñiga-Castillo et al. 2004; Rocha et al. 2005; Cromie 2009) (fig. 2). The genes are: *recJ*, *recF*, *recO* and *recR* (which initiate the repair of single-strand DNA breaks), *addA* and *addB* (which initiate the repair of double-strand DNA breaks), *recA* (which catalyzes strand exchange), *ruvA* and *ruvB* (which perform branch migration), *ruvC* (which resolves Holliday junction intermediates), and *priA* (which initiates replication during double-strand break repair). In addition, we selected *recG* whose protein function overlaps with that of the RuvAB complex (Meddows et al. 2004).

We analyzed these 12 genes in a diverse set of *Wolbachia* strains encompassing the four major supergroups A–D of *Wolbachia* diversity. We obtained sequences of HR genes by similarity searches using BLASTp (Altschul et al. 1990) against 20 sequenced *Wolbachia* genomes from supergroups A–D, as described in table 1. We also performed targeted resequencing of 14 additional *Wolbachia* strains from supergroups A and B (supplementary table S1, Supplementary Material online). Total DNA extraction, polymerase chain reaction (PCR), and sequencing were performed as previously described (Cordaux et al. 2008). In brief, DNA was extracted using a standard phenol–chlorophorm protocol, PCR was performed using the PCR primers and conditions provided in supplementary table S2, Supplementary Material online, and purified PCR products were directly sequenced on an ABI Prism 3130 Genetic Analyzer. The nucleotide sequences generated in this study have been deposited in GenBank under accession numbers KM066817–KM0066942.

**Table 1:** Distribution of 12 HR Genes in 20 *Wolbachia* Genomes

Super group	Strain	Host	Accession Number	*addA*	*addB*	*priA*	*recA*	*recF*	*recG*	*recJ*	*recO*	*recR*	*ruvA*	*ruvB*	*ruvC*	Reference
A	*w*Mel*	*Drosophila melanogaster*	AE017196	WD_0359	WD_0912	WD_1200	WD1050	WD_1286	WD_0824	WD_0312	WD_0219	WD1180	WD_1113	WD_1112	WD_0142	Wu et al. 2004
	*w*Ri*	*Drosophila simulans*	CP001391	WRi_004270	WRi_008710	WRi_011780	WRi_010830	WRi_013130	WRi_007900	WRi_004620	WRi_002090	WRi_011550	WRi_012870	WRi_012860	WRi_001110	Klasson et al. 2009
	*w*Ha*	*Drosophila simulans*	CP003884	wHa_04850	wHa_07710	wHa_10020	wHa_08760	wHa_10710	wHa_06960	wHa_05160	wHa_01890	wHa_09860	wHa_09320	wHa_09310	wHa_00600	Ellegaard et al. 2013
	*w*Uni	*Mudiscidifurax uniraptor*	ACFP00000000	M	F	F	F	F	F	F	F	M	F	F	F	Klasson et al. 2009
	*w*Wil	*Drosophila willistoni*	AAQP00000000	F	F	F	F	M	F	M	F	F	F	F	F	Remington et al. unpublished
	*w*Suzi	*Drosophila suzukii*	CAOU00000000	F	F	F	F	F	F	F	F	F	F	F	F	Siozios et al. 2013
B	*w*Pip-Pel*	*Culex quinquefasciatus*	AM999887	WP0175	WP0219	WP0766	WP0932	WP0901	WP0482	WP0630	WP0359	WP0769	WP0988	WP0987	WP1146	Klasson et al. 2008
	*w*AlbB	*Aedes albopictus*	CAGB00000000	F	F	F	F	F	F	F	F	F	F	F	F	Mavingui et al. 2012
	*w*Di	*Diaphorina citri*	AMZJ0000000	F	F	F	F	F	F	F	F	F	F	F	F	Saha et al. 2012
	*w*No*	*Drosophila simulans*	CP003883	wNo_09810	wNo_11150	wNo_04260	wNo_05950	wNo_05210	wNo_01520	wNo_03260	wNo_00850	wNo_04290	wNo_06840	wNo_06830	wNo_08060	Ellegaard et al. 2013
	*w*VitB	*Nasonia vitripennis*	AERW00000000	F	F	F	F	F	F	F	F	F	F	F	F	Kent et al. 2011
	*w*Bol1	*Hypolimnas bolina*	CAOH00000000	F	F	F	F	F	F	F	F	F	F	F	F	Duplouy et al. 2013
	*w*Pip-JHB	*Culex quinquefasciatus*	ABZA00000000	F	F	F	F	F	F	F	F	F	F	F	F	Salzberg et al. 2009
	*w*Pip-Mol	*Culex pipiens molestus*	CACK00000000	F	F	F	F	F	F	F	F	F	F	F	F	Sinkins et al. unpublished
C	*w*Oo*	*Onchocerca ochengi*	HE660029	wOo_08780	wOo_07360	wOo_05130	P	P	P	wOo_01160	P	P	P	P	P	Darby et al. 2012
	*w*Dir	*Dirofilaria immitis*	dirofilaria. nematod.es	F	F	P	P	P	P	F	P	P	P	P	P	Godel et al. 2012
D	*w*Bm*	*Brugia malayi*	AE017321	Wbm0172	Wbm0272	Wbm0745	Wbm0427	Wbm0126	Wbm0635	Wbm0124	Wbm0288	Wbm0746	Wbm0251	Wbm0252	Wbm0697	Foster et al. 2005
	*w*Wb	*Wucheria bancrofti*	ADHD00000000	F	M	Partial	F	M	Partial	F	M	M	M	M	M	Desjardins et al. 2013
	*w*Vol	*Onchocerca volvulus*	ADHE00000000	Partial	M	M	M	M	M	M	M	M	M	M	M	Desjardins et al. 2013
	wLs	*Litomosoides sigmodontis*	litomosoides. nematod.es	F	F	F	Partial	M	Partial	F	M	M	M	M	M	Comandatore et al. 2013

Note.—F, gene found (detected by BLASTp with start and stop codons); Partial, partial gene found (detected by BLASTp but lacking start or stop codon); P, pseudogene (only detected by BLASTn); M, missing gene (detected neither by BLASTp nor BLASTn). Locus tags are provided for the seven completed genomes (identified by *). The 14 other genomes are incomplete; therefore missing genes may be truly absent or may just be absent from the genome assembly.

### Sequence Analyses of *Wolbachia* Supergroups A and B

Nucleotide sequences of HR genes from 29 *Wolbachia* strains were aligned together by codons using the Muscle algorithm implemented in MEGA5 software (Tamura et al. 2011). We removed seven palindromic regions because they were difficult to align with confidence. These regions, resembling *Wolbachia* palindromic elements (WPE) (Ogata et al. 2005), were located in *addA* (nucleotide positions [np] 289–459, 907–1146, and 3076–3387), *addB* (np 778–1023 and np 2452–2655), and *priA* (np 523–1365 and 2587–2790). As these WPE-like sequences are inserted in frame and do not generate premature stop codons, they apparently do not inactivate the HR genes. The only exception is a*ddA* in the six *Wolbachia* strains from supergroup B hosted by isopods, in which a premature stop codon was generated at np 3100–3102, resulting in the deletion of the entire nuclease domain. This deletion may have limited functional consequences, as it has been shown that the loss of the nuclease domain significantly reduces efficiency of the exonuclease activity of the AddAB complex, but repair can still operate (Amundsen et al. 2009). Nevertheless, we conservatively split *addA* into two parts for our evolutionary analyses: a*ddA*_1 encompasses np 1–3099 and *addA*_2 encompasses np 3103–3837.

To avoid biased evolutionary analyses due to poor resolution, we removed all but one representative for all groups in which *Wolbachia* strains showed less than 0.2% pairwise nucleotide divergence across the 12 HR genes. This filter resulted in the removal of ten *Wolbachia* strains from analyses (supplementary table S3, Supplementary Material online). Our final data set then consisted of the 19 remaining *Wolbachia* strains.

Because intragenic recombination may alter phylogenetic and selection signals, we tested our data set for evidence of intragenic recombination using RDP3 (Martin et al. 2010), using seven different algorithms (rdp, geneconf, chimaera, maxchi, bootscan, siscan, and 3seq), linear sequences, and uninformative sequences masked. A recombination event was accepted when it was detected by at least four of the algorithms. Then, phylogenetic correlation of the recombinant was checked with the tree generated by RDP. After each confirmation, a rescan was launched. Intragenic recombination was detected in three regions: 1) np 1–346 of *ruvC* in strain *w*No, 2) np 2657–3051 of *addB* in strain *w*Di, and 3) np 1024–1122 of *addB* in all strains. These recombining sequences were removed from the analyses.

### Selection Analyses

Selection analyses were performed by calculating the ratios of the rates of nonsynonymous (Ka) to synonymous (Ks) nucleotide substitutions per site on each HR gene as implemented in codeml PAML4 software (Yang 2007). Ka/Ks ratios >1, <1, and =1 are indicative of positive selection, purifying selection, and neutral evolution, respectively. Pairs of models were compared using likelihood ratio tests (LRTs), as described in Yang (1998). When the *P* value of an LRT was significant at the 5% level, the model with the highest likelihood value was considered as the best-fit model. Otherwise, the two models were not significantly different, which means that adding complexity to the model does not improve its likelihood. As a result, the simplest model was considered as the best-fit model. We performed four successive types of comparisons.

First, we investigated global patterns of selection by comparing two models: 1) The neutral model with the Ka/Ks ratio forced to 1 in all branches of the phylogenetic tree (the simplest model in the comparison), and 2) a single Ka/Ks ratio model with the same Ka/Ks ratio in all branches.

**Fig. 3.—:**
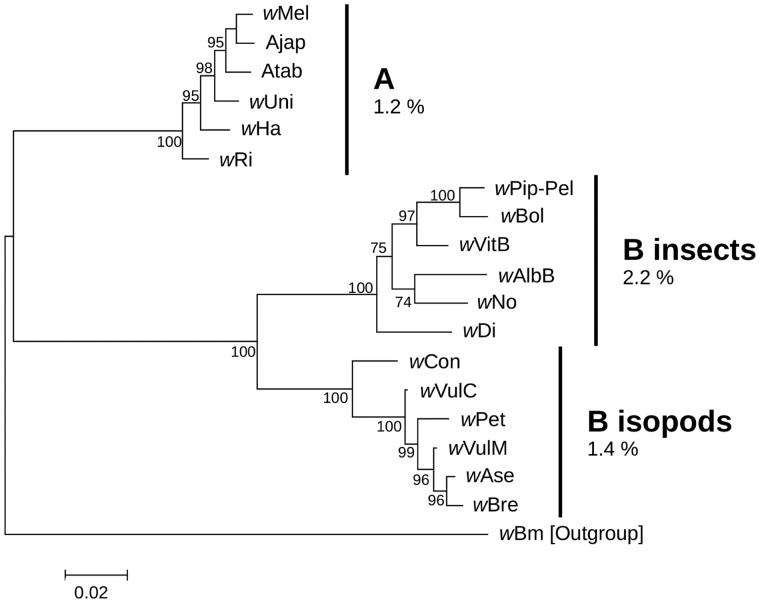
Maximum-likelihood phylogenetic tree of the 12 concatenated HR genes, based on the GTR+G model (gamma = 5) and 85% partial deletion. Bootstrap scores (%) are shown on branches (based on 1,000 replicates). The phylogenetic tree reveals three highly supported monophyletic groups, each composed of six *Wolbachia* strains including: Strains from supergroup A, from supergroup B hosted by insects and from supergroup B hosted by isopods. Intragroup mean distances are noted besides each group. Intergroup mean distances are: 10.2% between A and B from insects, 9.3% between A and B from isopods, and 5.9% between B from insects and B from isopods.

Second, for HR genes in which the single Ka/Ks ratio model was the best-fit model, we searched for selection heterogeneity by comparing two models: 1) The single Ka/Ks ratio model from the previous analysis (the simplest model in the comparison), and 2) a 3-Ka/Ks ratio model that implements a different Ka/Ks ratio for each of three monophyletic clusters (supergroup A, supergroup B hosted by insects, and supergroup B hosted by isopods; fig. 3).

Third, for HR genes in which the 3-Ka/Ks ratio model was the best-fit model, we searched for neutralization signal in the cluster with the highest Ka/Ks ratio in the 3-Ka/Ks ratio model by comparing two models: 1) the 3-Ka/Ks ratio model with the Ka/Ks ratio forced to 1 in the cluster with the highest Ka/Ks ratio in the 3-Ka/Ks ratio model (the simplest model in the comparison), and 2) the 3-Ka/Ks ratio model from the previous analysis.

Fourth, for the HR gene showing signs of neutralization, we searched for positive selection on codons (which may have inflated the Ka/Ks ratio) by using the branch-site model (Yang et al. 2005; Zhang et al. 2005).

To evaluate the robustness of our analyses, we used multiple phylogenetic frameworks based on a resampling strategy. Specifically, following a jackknife procedure, we removed each gene in turn from the concatenated alignment of the 12 HR genes and recalculated a phylogenetic tree based on the remaining genes. For each of the 12 alignments, we used MEGA5 to identify the best substitution model (GTR+G with gamma=5 in all cases). Next, each alignment was used to build a maximum-likelihood tree with 1,000 bootstrap replicates (85% partial deletion), using MEGA5. Hence, we obtained 12 phylogenetic trees, each of which was used as a phylogenetic hypothesis for selection analyses. The 12 trees corresponded to five different topologies exhibiting minor variations in terminal branching patterns (supplementary fig. S1, Supplementary Material online). This enabled us to calculate confidence intervals for LRTs.

## Results and Discussion

### Distribution of HR Genes in *Wolbachia* Strains

Analysis of the distribution of the 12 HR genes in 20 sequenced *Wolbachia* genomes from supergroups A–D (table 1) revealed that most of the HR genes from supergroup C *Wolbachia* strains were pseudogenized (Darby et al. 2012; Godel et al. 2012), indicating that the HR pathway has been inactivated in these strains. In addition, most of the HR genes were missing in all supergroup D strains except *w*Bm, which has an apparently intact HR pathway (Foster et al. 2005). Because *w*Bm is the only finished supergroup D genome (Foster et al. 2005), we cannot conclude whether some HR genes have not been sequenced or the HR pathway has been inactivated in other supergroup D genomes. In contrast, the HR pathway is apparently intact in *Wolbachia* strains from supergroups A and B (Wu et al. 2004; Klasson et al. 2008). The difference in completeness of the HR pathway or lack thereof is not really surprising given the contrast in host range (arthropods vs. nematodes for supergroups A/B and C/D, respectively) and lifestyle (reproductive parasite vs. mutualist for supergroups A/B and C/D, respectively) of these supergroups. Based on these observations, we focused our selection analyses on supergroups A and B. To obtain a more extensive sampling of HR genes in supergroups A and B, we performed targeted resequencing of 14 additional *Wolbachia* strains. After removal of strains showing less than 0.2% pairwise nucleotide divergence across the 12 HR genes (see Materials and Methods), our final data set consisted of 18 supergroups A and B *Wolbachia* strains and *w*Bm (supergroup D) as an outgroup.

### Selective Pressures on HR Genes

To measure the selective pressures acting on HR genes in supergroups A and B *Wolbachia* strains, we compared the Ka/Ks ratios of the 12 genes by using LRTs as implemented in PAML 4 (Yang 2007). To evaluate the robustness of our results, we performed analyses using multiple phylogenetic frameworks, based on a jackknife resampling strategy (see Materials and Methods). Assuming a single Ka/Ks ratio in each HR gene tree, the best-fit to the data was obtained for Ka/Ks ratios ranging from 0.05 (for *recA*) to 0.35 (for *addB*), which are all significantly less than 1 (all *P* < 0.001; table 2 and supplementary table S4, Supplementary Material online). This is indicative of purifying selection globally acting on all 12 HR genes. This strong conservation suggests that the HR pathway is evolutionarily important for *Wolbachia* strains from supergroups A and B. HR, together with repeats, creates high levels of genomic variation in prokaryotes (Rocha et al. 2005; Treangen et al. 2009). Strikingly, *Wolbachia* genomes from supergroups A and B bear an unusually high proportion of repeats and experience recombination (Werren and Bartos 2001; Kent and Bordenstein 2010; Cerveau, Leclercq, Leroy, et al. 2011; Leclercq et al. 2011; Ellegaard et al. 2013). Genome plasticity (and the factors favoring this plasticity) may represent an appreciable evolutionary advantage for *Wolbachia*, perhaps in relation with its ability to horizontally transfer between various arthropod host species (Cordaux et al. 2001) and with the fact that most of these strains are involved in an evolutionary arms race with their hosts (Cordaux et al. 2011). For example, it has been shown that recombination caused by repeats and HR genes enables some parasites to respond specifically to the adaptative immune system of the host (Finlay and Falkow 1997; Mehr and Seifert 1998).

**Table 2:** Summary of Selection Analysis for 12 HR Genes in 18 *Wolbachia* Strains

Gene	Best-Fit Model	*P* Value (Range)	Ka/Ks Values (Range)
*addA*_1	3 Ka/Ks	<0.02	A: 0.214–0.221; B insects: 0.277–0.291; B isopods: 0.175–0.193
*addA*_1	1 Ka/Ks	<0.001	0.187–0.199
*addB*	3 Ka/Ks	<0.001	A: 0.324–0.343; B insects: 0.431–0.447; B isopods: 0.382–0.395
*priA*	1 Ka/Ks	<0.001	0.143–0.147
*recA*	1 Ka/Ks	<0.001	0.049–0.050
*recF*	1 Ka/Ks	<0.001	0.225–0.239
*recG*	1 Ka/Ks	<0.001	0.128–0.136
*recJ*	1 Ka/Ks	<0.001	0.112–0.113
*recO*	3 Ka/Ks	<0.02	A: 0.294–0.307; B insects: 0.158–0.182; B isopods: 0.283–0.289
*recR*	1 Ka/Ks	<0.001	0.075–0.081
*ruvA*	Neutral foreground	0.066–0.094	A: 0.299–0.316; B insects: 0.211–0.216; B isopods: 1
*ruvB*	1 Ka/Ks	<0.001	0.072–0.074
*ruvC*	1 Ka/Ks	<0.001	0.218–0.221

Note.—Detailed results are provided in supplementary tables S4–S6, Supplementary Material online.

### Heterogeneity and Relaxation of Selection Pressures

To investigate whether purifying selection patterns have been stably maintained throughout *Wolbachia* evolution, we tested for heterogeneity in Ka/Ks ratios in each HR gene by comparing the above results to a model in which different Ka/Ks ratios were allowed in the three monophyletic clusters of the ingroup, each consisting of six *Wolbachia* strains: supergroup A, supergroup B hosted by insects, and supergroup B hosted by isopods (fig. 3). In this clustering scheme, average nucleotide divergence across the 12 HR genes ranged from 1% to 2% within groups and 6–10% between groups (fig. 3).

The model with three Ka/Ks ratios was significantly better than the model with a single Ka/Ks ratio under all 12 jackknifed phylogenetic hypotheses for four HR genes: *addA*_1, *addB*, *recO*, and *ruvA* (all *P* < 0.05; table 2 and supplementary table S5, Supplementary Material online). The 3-Ka/Ks ratio model was also better for *recF* and *ruvC* albeit for only a subset of jackknifed phylogenetic hypotheses*.* Given the limited robustness of the results for *recF* and *ruvC*, we conservatively rejected heterogeneity in Ka/Ks ratios for these two genes. In contrast, the results are robust for the four other genes *addA*_1, *addB*, *recO*, and *ruvA*, as all jackknifed trees consistently support heterogeneity in Ka/KS ratios. Heterogenous selection patterns have already been observed among different endosymbiont strains and were found to result from the effect of strong genetic drift or enhanced mutation rate (Wernegreen and Moran 1999; Itoh et al. 2002; Woolfit and Bromham 2003; Wernegreen and Funk 2004; Fry and Wernegreen 2005; Blanc et al. 2007), as expected in the context of genomic reduction undergone by *Wolbachia*.

To investigate whether heterogeneity in Ka/Ks ratios in these four HR genes may reflect relaxation of selective pressures leading to neutralization in a subset of the *Wolbachia* strains, we tested whether the highest Ka/Ks ratio in the 3-Ka/Ks ratio model was different from 1. We predicted that the elevated Ka/Ks ratio should be significantly different from 1 under relaxed selection without neutralization, and not significantly different from 1 under neutral evolution. For *addA*_1, *addB*, and *recO*, we found that the 3-Ka/Ks ratios model was significantly better than the model assuming neutralization in a subset of *Wolbachia* strains, under all 12 jackknifed phylogenetic hypotheses (all *P* < 0.01; table 2 and supplementary table S6, Supplementary Material online). In contrast, for *ruvA*, the two models were not significantly different from each other, implying that the highest Ka/Ks ratio (0.44) in the 3-Ka/Ks ratio model is not significantly different from 1. This result was robust, as it was supported by all 12 jackknifed phylogenetic hypotheses (*P* ranging from 0.066 to 0.094). In other words, these results indicate relaxed selection leading to neutralization in *ruvA* in supergroup B *Wolbachia* strains from isopods. To test whether the neutralization signal was specific to the isopod *Wolbachia* group or also characterized the other *Wolbachia* groups, we tested whether there was a significant difference when assuming a Ka/Ks ratio of 1 for any of the other two groups (i.e., A supergroup strains and B supergroup strains from insects). In both cases, neutral evolution was rejected (supplementary table S6, Supplementary Material online), indicating that the signal of neutralization in *ruvA* specifically applies to supergroup B *Wolbachia* strains from isopods.

To substantiate these results, we further analyzed *ruvA* molecular evolution in *Wolbachia* strains of isopods. Average genetic distance between isopod *Wolbachia* strains for *ruvA* was 1.2% (range 0.2–2.0%) (supplementary fig. S1, Supplementary Material online). There was also no obvious sign of pseudogenization in *ruvA*, as it does not contain any inactivating mutation (i.e., frameshift or premature stop codon) or homopolymeric tract of poly(A) (>9 bp) which could induce slippage of RNA polymerase (Tamas et al. 2008). In addition, to test whether positive selection, rather than neutral evolution, may have inflated the Ka/Ks ratio, we used the branch-site model of PAML to search for positively selected codons in *ruvA* (Yang et al. 2005; Zhang et al. 2005). No codon was found to be under positive selection (*P* = 0.71). Therefore, there is no evidence to support an effect of positive selection in our results.

To further investigate the evolutionary history of *ruvA* in supergroup B *Wolbachia* strains from isopods, we searched for potential ancestral substitutions specific to isopod *Wolbachia* strains that might have contributed to impair RuvA functional efficiency and, possibly, triggered gene neutralization. Inspection of the *ruvA* sequence alignment revealed 14 nucleotide substitutions exclusively shared by isopod *Wolbachia* strains, all located in functional domains, eight of which being nonsynonymous substitutions (table 3). Interestingly, five of these amino acid changes modify the physicochemical properties of the RuvA protein, in terms of charge, hydrophobicity or aliphatic property. Unfortunately, *Wolbachia* endosymbionts are unculturable bacteria. Therefore, the actual consequences of these five amino acid changes on RuvA functionality cannot be directly tested. Nevertheless, to hint at the potential functional consequences of these amino acid changes, we reviewed the literature and collated a list of 51 amino acid sites in the approximately 200 amino acid-long RuvA protein that are considered important for proper folding, multimerization or DNA binding, based on crystallography (Rafferty et al. 1996; Nishino et al. 1998, 2000; Roe et al. 1998; Ariyoshi et al. 2000; Yamada et al. 2002; Prabu et al. 2006, 2009) or mutagenesis studies (Nishino et al. 1998; Privezentzev et al. 2005; Baharoglu et al. 2008; Fujiwara et al. 2008; Le Masson et al. 2008; Mayanagi et al. 2008; Bradley et al. 2011). We found that one amino acid change inducing a physicochemical modification in isopod *Wolbachia* RuvA falls in this list of 51 important amino acids (Lys-118). In *Escherichia coli*, Lys-118 plays an important role in DNA binding through nonpolar interaction with its aliphatic chain (Ariyoshi et al. 2000). In all supergroup A and supergroup B *Wolbachia* strains from insects, Lys is replaced by Leu, but this change is not expected to impair function as Leu is also an aliphatic amino acid. In contrast, in the ancestor of all supergroup B *Wolbachia* strains from isopods, the aliphatic Leu was replaced by the nonlinear aliphatic Pro, which may have altered RuvA DNA-binding activity. Strikingly, in the *w*Ase isopod *Wolbachia* strain, an additional nonsynonymous substitution resulted in the replacement of Pro by Ser (which is a nonaliphatic amino acid). We speculate that this substitution was not eliminated by purifying selection because the ancestral Pro had already contributed to impair RuvA function in isopod *Wolbachia* strains. If so, Leu-118-Pro might have played a key role in *ruvA* neutralization in these *Wolbachia* strains.

**Table 3:** Nucleotide Substitutions of *ruvA* Specific to B Supergroup *Wolbachia* Strains from Isopods

Substitution Type	Nucleotide Substitution	Functional Domain^a^	Amino Acid Substitution	Amino Acid Property Change
NS	G70A	I	V24I	Neutral > positive
	T76C	I	Y26H		
	G125A	I	S42N		
	T353C	II	L118P^b^	Aliphatic >
				nonaliphatic
	G493A^c^	III	D165N	Negative >
				neutral
	T497C^c^	III	T166M	Nonhydrophobic >
				hydrophobic
	C524A^c^	III	P175Q	Hydrophobic >
				nonhydrophobic
	A539G	III	K180R	
S	T87C	I		
	T280C	II		
	A357G	II		
	T457C	III		
	A498G	III		
	A513G^c^	III		

^a^Domains I and II are implicated in tetramerization of the protein and junction-DNA binding; domain III is implicated in branch migration through heteroduplex contact with RuvB.

^b^S in *w*Ase due to an additional mutation at np 352.

^c^Except for *w*Con.

Although we cannot formally ascertain which (if any) of the five amino acid changes may have been primordial in the initiation of the *ruvA* neutralization process in isopod *Wolbachia* strains, a plausible evolutionary scenario is that a nonsynonymous substitution occurred in the ancestral *ruvA* gene of isopod *Wolbachia* that significantly affected RuvA functionality (possibly the one leading to Leu-118-Pro). RuvA is part of the RuvAB complex, which performs branch migration during the HR process. Interestingly, RuvAB and RecG functions overlap (Meddows et al. 2004) and our results indicate that RecG has been consistently evolving under strong purifying selection throughout supergroups A and B *Wolbachia* evolution (Ka/Ks = 0.13; *P* < 0.001). Therefore, the impaired RuvA function may have resulted in a decreased efficiency of the RuvAB complex, which may have been compensated by RecG in the isopod *Wolbachia* ancestor, as part of a process of nonorthologous gene displacement (Koonin et al. 1996). As soon as RuvA function was impaired, gene sequence could start undergoing independent neutral evolution in each lineage derived from this ancestor (table 3), thereby reinforcing functional impairment and gene neutralization. As a result, a pair of genes with redundant functions is now experiencing contrasting selective pressures in *Wolbachia* strains from isopods, with *recG* experiencing strong purifying selection, whereas *ruvA* has been neutralized. In support to our inferences, it is noteworthy that nonorthologous gene displacement has already been documented for recombination functions, as exemplified by the substitution of *ruvC* by *recU* or *recBCD* by *addAB* (Ayora et al. 2004; Rocha et al. 2005; Cromie 2009).

## Conclusion

In the context of genomic reduction undergone by *Wolbachia* genomes, redundancy is expected to be lost through the process of pseudogenization (Ogata et al. 2001; Silva et al. 2001; Moya et al. 2008). Numerous reports have focused on global pseudogenization events affecting the overall repertoire of genes of a genome due to enhanced genetic drift and less effective purifying selection (Moran 1996; Itoh et al. 2002; Woolfit and Bromham 2003; Fuxelius et al. 2008; McCutcheon and Moran 2012). In contrast, studies reporting relaxation of selection targeting redundant genes are scarce. This has been reported in the case of the functional replacement of *B. aphidicola* BCc by *S. symbiotica* in the host *C. cedri* (Pérez-Brocal et al. 2006). However, in this example, redundancy occurs between two different but closely interacting genomes, whereas we report a case of probable gene neutralization in the context of functional redundancy within a single genome.

Our results provide empirical evidence to support that relaxation of selection on specific genes and genetic drift act in synergy with the process of Muller’s ratchet (Moran 1996; McCutcheon and Moran 2012). It is even plausible that genetic drift triggers specific relaxation of selection while slightly and mildly deleterious mutations are accumulating. In the case of redundant genes, these mutations would render the gene less “efficient” in its function in comparison with the analogous gene. As a result, the analogous gene would be favored to fulfill the function relative to the gene affected by slightly deleterious mutations and/or some compensatory measures would be set up. On the long-term, this would render the gene nonessential and thus initiate the process of specific neutralization, ultimately leading to pseudogenization and complete loss. In any event, targeted neutralization of *RuvA* indicates that genomic reduction is an ongoing process in *Wolbachia* endosymbionts.

## Supplementary Material

Supplementary figure S1 and tables S1–S6 are available at *Genome Biology and Evolution* online (http://www.gbe.oxfordjournals.org/).

Supplementary Data
