# Supplementary material for: Signs of Neutralization in a Redundant Gene Involved in Homologous Recombination in Wolbachia Endosymbionts
Source: Genome Biol Evol. 2014 Sep 17;6(10):2654–64. doi: 10.1093/gbe/evu207 (PMC4224334; doi:10.1093/gbe/evu207)
Supplement: Supplementary Data [file supp_6_10_2654__index.html]

Signs of Neutralization in a Redundant Gene Involved in Homologous Recombination in Wolbachia Endosymbionts — Supplementary Data 

# Signs of Neutralization in a Redundant Gene Involved in Homologous Recombination in *Wolbachia* Endosymbionts

## Supplementary Data

files

**Files in this Data Supplement:**

- Supplementary Data - pdf file
- Supplementary Data - xls file
- Supplementary Data - xls file
- Supplementary Data - xls file
- Supplementary Data - xls file
- Supplementary Data - xls file
- Supplementary Data - xls file
