# Supplementary figures and images for: Signs of Neutralization in a Redundant Gene Involved in Homologous Recombination in Wolbachia Endosymbionts
Source: Genome Biol Evol. 2014 Sep 17;6(10):2654–64. doi: 10.1093/gbe/evu207 (PMC4224334; doi:10.1093/gbe/evu207)

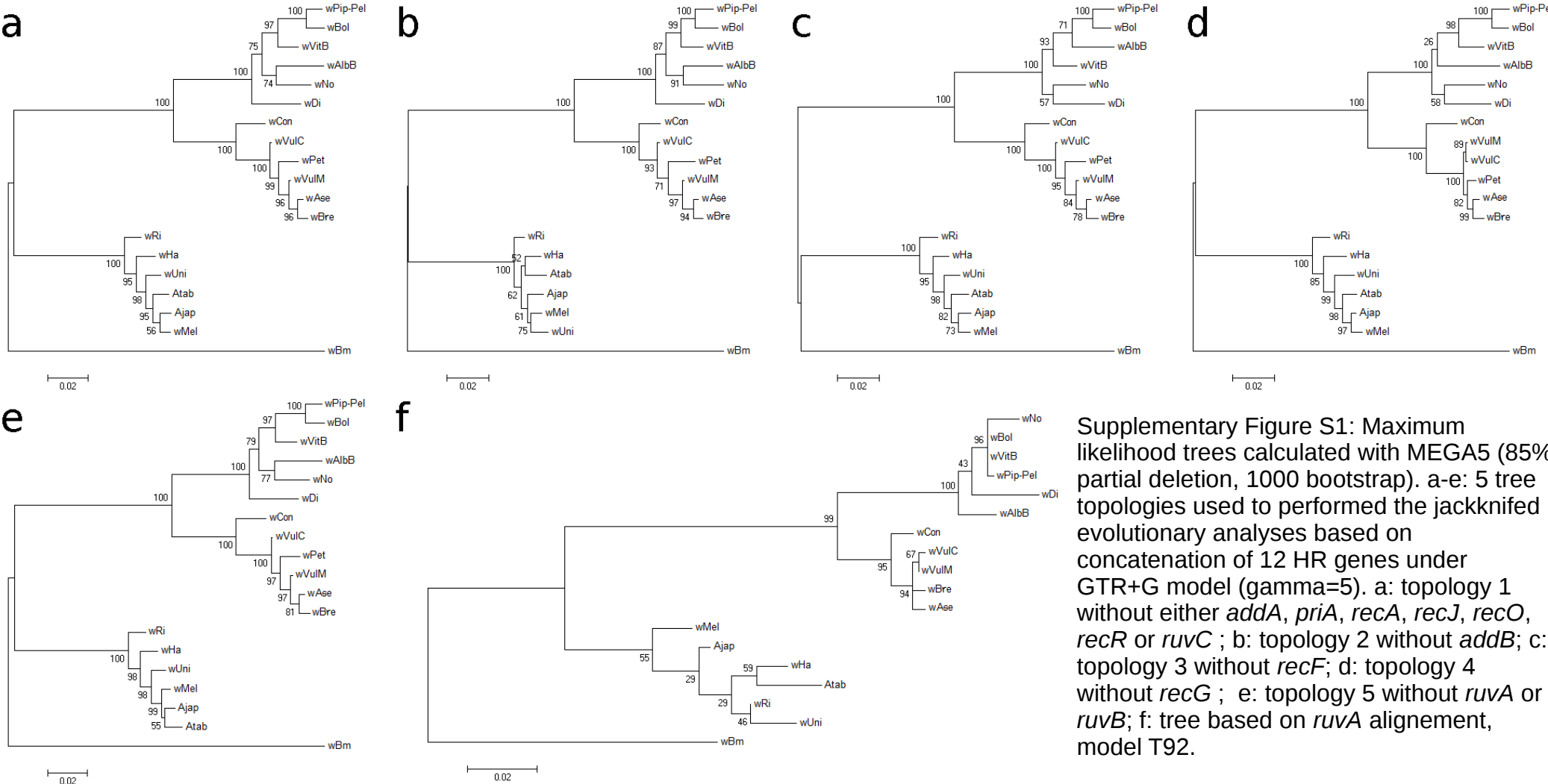

Supplement: Supplementary Data [file supp_evu207_SupplFig_S1.pdf]
